# Treatments for Biomedical Abnormalities Associated with Autism Spectrum Disorder

**DOI:** 10.3389/fped.2014.00066

**Published:** 2014-06-27

**Authors:** Richard Eugene Frye, Daniel A. Rossignol

**Affiliations:** ^1^Department of Pediatrics, Arkansas Children’s Hospital Research Institute, University of Arkansas for Medical Sciences, Little Rock, AR, USA; ^2^Rossignol Medical Center, Irvine, CA, USA

**Keywords:** autism spectrum disorders, mitochondria, folate receptor alpha, folinic acid, folate metabolism, redox regulation, oxidative stress, tetrahydrobiopterin

## Abstract

Recent studies point to the effectiveness of novel treatments that address physiological abnormalities associated with autism spectrum disorder (ASD). This is significant because safe and effective treatments for ASD remain limited. These physiological abnormalities as well as studies addressing treatments of these abnormalities are reviewed in this article. Treatments commonly used to treat mitochondrial disease have been found to improve both core and associated ASD symptoms. Double-blind, placebo-controlled (DBPC) studies have investigated l-carnitine and a multivitamin containing B vitamins, antioxidants, vitamin E, and co-enzyme Q10 while non-blinded studies have investigated ubiquinol. Controlled and uncontrolled studies using folinic acid, a reduced form of folate, have reported marked improvements in core and associated ASD symptoms in some children with ASD and folate related pathway abnormities. Treatments that could address redox metabolism abnormalities include methylcobalamin with and without folinic acid in open-label studies and vitamin C and *N*-acetyl-l-cysteine in DBPC studies. These studies have reported improved core and associated ASD symptoms with these treatments. Lastly, both open-label and DBPC studies have reported improvements in core and associated ASD symptoms with tetrahydrobiopterin. Overall, these treatments were generally well-tolerated without significant adverse effects for most children, although we review the reported adverse effects in detail. This review provides evidence for potentially safe and effective treatments for core and associated symptoms of ASD that target underlying known physiological abnormalities associated with ASD. Further research is needed to define subgroups of children with ASD in which these treatments may be most effective as well as confirm their efficacy in DBPC, large-scale multicenter studies.

## Background

The autism spectrum disorders (ASD) are a group of behaviorally defined neurodevelopmental disorders with lifelong consequences. They are defined by impairments in communication and social interaction along with restrictive and repetitive behaviors (1). The definition of ASD has recently undergone revision. Previously, the Diagnostic Statistical Manual (DSM) Version IV Text Revision divided ASD into several diagnoses including autistic disorder, Asperger syndrome, and pervasive developmental disorder-not otherwise specified. The new revision of the DSM now does not differentiate between these ASD subtypes and considers communication and social impairments together in one symptom class (2). Complicating this change is the fact that over the past several decades, most research has used a framework from the former DSM versions.

Autism spectrum disorder has been recently estimated to affect 1 out of 68 individuals in the United States (3) with four times more males than females being affected (4). Over the past two decades, the prevalence of the ASDs has grown dramatically, although the reasons for this increase are continually debated. Despite decades of research on ASD, identification of the causes of and treatments for ASD remain limited. The standard-of-care treatment for ASD is behavioral therapy that requires full-time engagement of a one-on-one therapist typically requiring many years of treatment, and recent reviews have pointed out that controlled studies on commonly used behavior therapies are generally lacking (5). The only medical treatments approved by the United States of America Food and Drug Administration for ASD are antipsychotic medications. However, these medications only treat a symptom associated with ASD, irritability, but not any core ASD symptom. In children, these medications can be associated with significant adverse effects, including detrimental changes in body weight as well as triglyceride, cholesterol, and blood glucose concentrations within a short time (6) and they also increase the risk of type 2 diabetes (7). In some studies, the percentage of children experiencing these side effects is quite high. For example, one recent study reported that 87% of ASD children had side effects with risperidone, including drowsiness, weight gain, and rhinorrhea (8).

A great majority of ASD research has concentrated on genetic causes of ASD (9) despite the fact that inherited single gene and chromosomal defects are only found in the minority of cases (10). In fact, several recent studies that have conducted genome wide searches for common genetic defects across large samples of ASD children have only identified rare *de novo* mutations, thereby pointing to acquired mutations and/or mutations secondary to errors in DNA maintenance rather than inherited genetic syndromes (11, 12). As research in the field of ASD continues, it is becoming clear that the etiology of most ASD cases involves complicated interactions between genetic predisposition and environmental exposures or triggers. Indeed, a recent study of dizygotic twins estimated that the environment contributes a greater percentage of the risk of developing autistic disorder as compared to genetic factors (13). Another study of over two million children reported that environmental risk factors accounted for approximately 50% of ASD risk (14). Recent reviews have outlined the many environmental factors that are associated with ASD and have described how polymorphisms in specific genes can combine with the environment to cause neurodevelopmental problems (15).

Recent studies have suggested that ASD is associated with impairments in basic physiological processes such as redox (16) and mitochondrial (9) metabolism as well as abnormalities in regulating essential metabolites such as folate (17), tetrahydrobiopterin (18–20), glutathione (21–23), cholesterol (24), carnitine (25–28), and branch chain amino acids (29). Although many of these studies have based their findings on peripheral markers of abnormal metabolism, many studies have documented some of these same abnormalities in the brain of individuals with ASD, including mitochondrial dysfunction and oxidative stress (30) and one study has demonstrated a link between oxidative stress, inflammation, and mitochondrial dysfunction in the brain of individuals with ASD (23). Interestingly, several of these physiological abnormalities are also observed in genetic syndromes associated with ASD. For example, mitochondrial dysfunction is prevalent in both idiopathic ASD (31) and is associated with Rett syndrome (32–34), PTEN mutations (35), Phelan-McDermid syndrome (36), 15q11-q13 duplication syndrome (37, 38), Angelman syndrome (39), Septo-optic dysplasia (40), and Down syndrome (41, 42).

**Figure 1:**
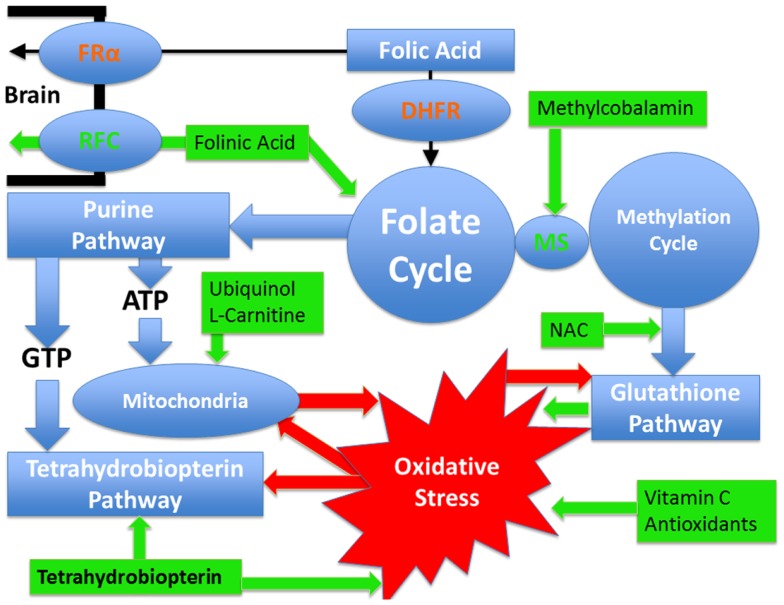
**Pathways affected in autism spectrum disorder that are discussed in this article as well as the treatments discussed with their points of action**. Pathways are outlined in blue while treatments are outlined in green. Oxidative stress is outlined in red and the red arrows demonstrate how it can negatively influence metabolic pathways. Certain pathways such as glutathione and tetrahydrobiopterin pathways have an antioxidant effect and a reciprocal relationship with oxidative stress such that they can improve oxidative stress but at the same time oxidative stress has a direct detrimental effect on them. Mitochondrial dysfunction and oxidative stress have mutually negative effects on each other such that oxidative stress causes mitochondrial dysfunction while mitochondrial dysfunction worsens oxidative stress. Dihydrofolate reductase (DHFR) is colored in red since polymorphisms in this gene, that are commonly seen in individuals with autism, have a detrimental effect on the reduction of folic acid such that the entry of folic acid into the folate cycle is decreased. Folinic acid enters the folate cycle without requiring this enzyme. Similarly the folate receptor alpha can be impaired in individuals with autism by autoantibodies and by mitochondrial dysfunction. In such cases, folinic acid can cross the blood–brain barrier by the reduced folate carrier. Methionine synthase (MS) connects the folate and methylation cycles and requires methylcobalamin as a cofactor.

Identifying the metabolic or physiological abnormalities associated with ASD is important, as treatments for such abnormalities may be possible. Thus, a better understanding of these abnormalities may allow for the development of novel treatments for children with ASD. Below the evidence for metabolic abnormalities related to ASD that may be amenable to treatment are discussed along with the evidence of potential treatments for these disorders. Figure 1 provides a summary of the pathways and demonstrates which pathways are targeted by the better studied treatments. In addition, a section on the common adverse effects of these treatments follows the discussion of treatments.

## Review of Treatable Conditions and Their Potential Treatments

### Mitochondrial dysfunction

Recent studies suggested that 30–50% of children with ASD possess biomarkers consistent with mitochondrial dysfunction (31, 43) and that the prevalence of abnormal mitochondrial function in immune cells derived from children with ASD is exceedingly high (44, 45). Mitochondrial dysfunction has been demonstrated in the postmortem ASD brain (23, 30, 46–49) and in animal models of ASD (50). Novel types of mitochondrial dysfunction have been described in children with ASD (28, 51, 52) and in cell lines derived from children with ASD (53, 54). Several studies suggest that children with ASD and mitochondrial dysfunction have more severe behavioral and cognitive disabilities compared with children who have ASD but without mitochondrial dysfunction (55–57). Interestingly, a recent review of all of the known published cases of mitochondrial disease and ASD demonstrated that only about 25% had a known genetic mutation that could account for their mitochondrial disease (31).

Treatments that are typically used for patients with mitochondrial disease have been shown to improve functioning in some children with ASD (31). Several studies, including two double-blind, placebo-controlled (DBPC) studies (58, 59) and case reports (25, 37, 60–63) have reported improvements in core and associated ASD behaviors with l-carnitine treatment. Two DBPC studies using a multivitamin containing B vitamins, antioxidants, vitamin E, and co-enzyme Q10 reported various improvements in ASD symptoms compared to placebo (64, 65). Several other antioxidants (66), including vitamin C (67), methylcobalamin (68–70), *N*-acetyl-l-cysteine (71–73), ubiquinol (74), and carnosine (75), have also reported to demonstrate significant improvements in ASD behaviors and may function to improve mitochondrial function.

Thus, many treatments that are believed to improve mitochondrial function have been shown to be helpful for some children with ASD. However, none of these studies have specifically selected children with mitochondrial dysfunction or disease to study, so it is difficult to know if individuals with ASD and mitochondrial dysfunction would benefit the most from these treatments or whether these treatments are effective for a wider group of children with ASD. One study did demonstrate that the multivitamin used for treatment resulted in improvements in biomarkers of energy metabolism (as well as oxidative stress) suggesting that the effect of the multivitamin may have been at least partially related to improvements in mitochondrial function (65). Clearly, this is a fertile area for research but there remain several complications that could impede moving forward in a systematic way. For example, given the inconsistency in the prevalence estimates of mitochondrial disease and dysfunction across studies (ranging from about 5–80%), the notion that mitochondrial abnormalities are even associated with ASD is somewhat controversial. This may be, in part, due to the unclear distinction between mitochondrial disease and dysfunction. However, even the lower bound of the prevalence estimate of 5% is significant, as mitochondrial disease is only believed to affect <0.1% of individuals in the general population and given the current high prevalence of ASD, a disorder that affects even 5% of individuals with ASD would add up to millions of individuals who have the potential to have a treatable metabolic abnormality. Other complicating factors include the fact that there are many treatments for mitochondrial disease and these treatments have not been well-studied (76). Hopefully, the increased interest in treatments for mitochondrial disease will help improve our knowledge of how to best treat mitochondrial disease so that such information can be applied to children who have mitochondrial disease and dysfunction with ASD. Other recent approaches include the *in vitro* assessment of compounds that may improve mitochondrial function in individuals with ASD (53).

### Folate metabolism

Several lines of evidence point to abnormalities in folate metabolism in ASD. Several genetic polymorphisms in key enzymes in the folate pathway have been associated with ASD. These abnormalities can cause decreased production of 5-methyltetrahydrofolate, impair the production of folate cycle metabolites and decrease folate transport across the blood–brain barrier and into neurons. Indeed, genetic polymorphisms in methylenetetrahydrofolate reductase (22, 77–85), dihydrofolate reductase (86) and the reduced folate carrier (22) have been associated with ASD.

Perhaps the most significant abnormalities in folate metabolism associated with ASD are autoantibodies to the folate receptor alpha (FRα). Folate is transported across the blood–brain barrier by an energy-dependent receptor-mediated system that utilizes the FRα (87). Autoantibodies can bind to the FRα and greatly impair its function. These autoantibodies have been linked to cerebral folate deficiency (CFD). Many cases of CFD carry a diagnosis of ASD (88–94) and other individuals with CFD are diagnosed with Rett syndrome, a disorder closely related to ASD within the pervasive developmental disorder spectrum (95–97). Given that the FRα folate transport system is energy-dependent and consumes ATP, it is not surprising that a wide variety of mitochondrial diseases (91, 94, 97–102) and novel forms of mitochondrial dysfunction related to ASD (52) have been associated with CFD. Recently, Frye et al. (17) reported that 60% and 44% of 93 children with ASD were positive for the blocking and binding FRα autoantibody, respectively. This high rate of FRα autoantibody positivity was confirmed by Ramaekers et al. (103) who compared 75 ASD children to 30 non-autistic controls with developmental delay. The blocking FRα autoantibody was positive in 47% of children with ASD but in only 3% of the control children.

Many children with ASD and CFD have marked improvements in clinical status when treated with folinic acid – a reduced form of folate that can cross the blood–brain barrier using the reduced folate carrier rather than the FRα transport system. Several case reports (89) and case series (90, 91) have described neurological, behavioral, and cognitive improvements in children with documented CFD and ASD. One case series of five children with CFD and low-functioning autism with neurological deficits found complete recovery from ASD symptoms with the use of folinic acid in one child and substantial improvements in communication in two other children (90). In another study of 23 children with low-functioning regressive ASD and CFD, 2 younger children demonstrated full recovery from ASD and neurological symptoms, 3 older children demonstrated improvements in neurological deficits but not in ASD symptoms, and the remainder demonstrated improvements in neurological symptoms and partial improvements in some ASD symptoms with folinic acid; the most prominent improvement was in communication (91). Recently, in a controlled open-label study, Frye et al. (17) demonstrated that ASD children who were positive for at least one of the FRα autoantibodies experienced significant improvements in verbal communication, receptive and expressive language, attention, and stereotypical behavior with high-dose (2 mg/kg/day in two divided doses; maximum 50 mg/day) folinic acid treatment with very few adverse effects reported.

Thus, there are several lines of converging evidence suggesting that abnormalities in folate metabolism are associated with ASD. Evidence for treatment of these disorders is somewhat limited but it is growing. For example, treatment studies have mostly concentrated on the subset of children with ASD who also possess the FRα autoantibodies. These studies have only examined one form of reduced folate, folinic acid, and have only examined treatment response in limited studies. Thus, large DBPC studies would be very helpful for documenting efficacy of this potentially safe and effective treatment. In addition, the role of other abnormalities in the folate pathway beside FRα autoantibodies, such as genetic polymorphisms, in treatment response needs to be investigated. It might also be important to investigate the role of treatment with other forms of folate besides folinic acid, but it might also be wise to concentrate research on one particular form of folate for the time being so as to optimize the generalizability of research studies in order to have a more solid understanding of the role of folate metabolism in ASD. Given the ubiquitous role of folate in many metabolic pathways and the fact that it has a role in preventing ASD during the preconception and prenatal periods (104), this line of research has significant potential for being a novel treatment for many children with ASD.

### Redox metabolism

Several lines of evidence support the notion that some children with ASD have abnormal redox metabolism. Two case-control studies have reported that redox metabolism in children with ASD is abnormal compared to unaffected control children (22, 105). This includes a significant decrease in reduced glutathione (GSH), the major intracellular antioxidant, and mechanism for detoxification, as well as a significant increase in the oxidized disulfide form of glutathione (GSSG). The notion that abnormal glutathione metabolism could lead to oxidative damage is consistent with studies which demonstrate oxidative damage to proteins and DNA in peripheral blood mononuclear cells and postmortem brain from ASD individuals (23, 30, 106), particularly in cortical regions associated with speech, emotion, and social behavior (30, 107).

Treatments for oxidative stress have been shown to be of benefit for children with ASD. In children with ASD, studies have demonstrated that glutathione metabolism can be improved with subcutaneously injected methylcobalamin and oral folinic acid (69, 105), a vitamin and mineral supplement that includes antioxidants, co-enzyme Q10, and B vitamins (65) and tetrahydrobiopterin (20). Interestingly, recent DBPC studies have demonstrated that *N*-acetyl-l-cysteine, a supplement that provides a precursor to glutathione, was effective in improving symptoms and behaviors associated with ASD (72, 73). However, glutathione was not measured in these two studies.

Small (64, 67), medium (72, 73), and large (108) sized DPBC trials and small and medium-sized open-label clinical trials (68, 70) demonstrate that novel treatments for children with ASD, which can address oxidative stress are associated with improvements in core ASD symptoms (68, 70, 72), sleep and gastrointestinal symptoms (64), hyperactivity, tantruming, and parental impression of general functioning (108), sensory-motor symptoms (67), and irritability (72, 73). These novel treatments include *N*-acetyl-l-cysteine (72, 73), methylcobalamin with (69, 70) and without (68) oral folinic acid, vitamin C (67), and a vitamin and mineral supplement that includes antioxidants, co-enzyme Q10, and B vitamins (64, 65).

Several other treatments that have antioxidant properties (66), including carnosine (75), have also been reported to significantly improve ASD behaviors, suggesting that treatment of oxidative stress could be beneficial for children with ASD. Many antioxidants can also help improve mitochondrial function (31), suggesting that clinical improvements with antioxidants may occur through a reduction of oxidative stress and/or an improvement in mitochondrial function.

These studies suggest that treatments that address oxidative stress may improve core and associated symptoms of ASD. Furthermore, these treatments are generally regarded as safe with a low prevalence of adverse effects. Unfortunately many studies that have looked at antioxidants and treatments that potentially support the redox pathway did not use biomarkers to measure redox metabolism status in the participants or the effect of treatment on redox pathways. Including biomarkers in future studies could provide important information regarding which patients may respond to treatments that address redox metabolism and can help identify the most effective treatments. Since there are many treatments used to address oxidative stress and redox metabolism abnormalities in clinical practice and in research studies, the most effective treatments need to be carefully studied in DBPC studies to document their efficacy and effectiveness. Overall, the treatments discussed above have shown some promising results and deserve further study.

### Tetrahydrobiopterin metabolism

Tetrahydrobiopterin (BH_4_) is a naturally occurring molecule that is an essential cofactor for several critical metabolic pathways, including those responsible for the production of monoamine neurotransmitters, the breakdown of phenylalanine, and the production of nitric oxide (19). BH_4_ is readily oxidized by reactive species, leading it to be destroyed in the disorders where oxidative stress is prominent such as ASD (18). Abnormalities in several BH_4_ related metabolic pathways or in the products of these pathways have been noted in some individuals with ASD, and the cerebrospinal fluid concentration of BH_4_ has been reported to be depressed in some individuals with ASD (19). Clinical trials conducted over the past 25 years have reported encouraging results using sapropterin, a synthetic form of BH_4_, to treat children with ASD (19). Three controlled (109–111) and several open-label trials have documented improvements in communication, cognitive ability, adaptability, social abilities, and verbal expression with sapropterin treatment in ASD, especially in children younger than 5 years of age and in those who are relatively higher functioning at the beginning of the trial (19).

Frye has shown that the ratio of serum citrulline-to-methionine is related to the BH_4_ concentration in the cerebrospinal fluid, suggesting that abnormalities in both oxidative stress and nitric oxide metabolism may be related to central BH_4_ deficiency (18). More recently, Frye et al. demonstrated, in an open-label study, that sapropterin treatment improves redox metabolism and fundamentally alters BH_4_ metabolism in children with ASD. Interestingly, serum biomarkers of nitric oxide metabolism were found to predict response to sapropterin treatment in children with ASD (20), thereby suggesting that the therapeutic effect of BH_4_ supplementation may be specific to its effect on nitric oxide metabolism.

The potential positive effects on nitric oxide metabolism by BH_4_ supplementation could be significant for several reasons. The literature supports an association between ASD and abnormalities in nitric oxide metabolism. Indeed studies have documented alterations in nitric oxide synthase genes in children with ASD (112, 113). In the context of low BH_4_ concentrations, nitric oxide synthase produces peroxynitrite, an unstable reactive nitrogen species that can result in oxidative cellular damage. Indeed, nitrotyrosine, a biomarker of reactive nitrogen species, has been shown to be increased in multiple tissues in children with ASD, including the brain (22, 23, 107, 114, 115). Thus, BH_4_ supplementation could help stabilize nitric oxide synthase as well as act as an antioxidant and improve monoamine neurotransmitter production. Further DBPC studies using biomarkers of metabolic pathways related to BH_4_ metabolism will be needed to determine which children with ASD will most benefit from formulations of BH_4_ supplementation like sapropterin.

## Potential Adverse Effects

Although many of the treatments discussed within this manuscript are considered safe and are generally well-tolerated, it is important to understand that these treatments are not without potential adverse effects. In general, these treatments are without serious adverse effects but some children may not tolerate all treatments well. Systematic and controlled studies are best at providing data on adverse effects, so the true adverse effects of the supplements discussed will only be based on the limited treatments that have been studied in such a fashion. It is also important to understand that because of the complicated nature of the effects of these treatments, they should only be used under the care of a medical professional with appropriate expertise and experience.

Controlled studies for treatments that address mitochondrial disorders include l-carnitine and a multivitamin with various mitochondrial supplements. In one small DBPC study, there were no significant adverse events reported in the 16 children treated with l-carnitine (59) while a second small DBPC trial reported no differences between the adverse effects reported by the treatment and placebo groups; notably, more patients in the placebo group withdrew from the study because of adverse effects (58). Thus, there is no data to suggest that l-carnitine has any significant adverse effects. In the large DBPC multivitamin study, about equal numbers of children in the treatment and placebo groups withdrew from the study because of behavior or gastrointestinal issues (65). In another small DBPC study, the investigators noted that two children began to have nausea and emesis when they started receiving the treatment at nighttime on an empty stomach (64). This adverse effect resolved when the timing of the treatment was adjusted. Thus, with proper dosing of this multivitamin, it appears rather safe and well-tolerated.

Controlled studies for folate pathway abnormalities only include folinic acid. In a medium-sized, open-label controlled study, 44 children with ASD and the FRα autoantibody were treated with high-dose folinic acid (2 mg/kg/day in two divided doses; maximum 50 mg/day) and four children discontinued the treatment because of an adverse effect (17). Of the four children who discontinued the treatment, three children, all being concurrently treated with risperidone, demonstrated increased irritability soon after starting the high-dose folinic acid while the other child experienced increased insomnia and gastroesophageal reflux after 6 weeks of treatment. Since there was no placebo in this study, the significance of these adverse effects is difficult to determine. For example, it is not clear whether this was related to concurrent risperidone treatment or was related to a baseline high irritability resulting in the needed for risperidone. All other participants completed the trial without significant adverse effects. Due to the timing of the adverse events in the children on risperidone in this trial, to be safe, the authors suggested caution when using folinic acid in children already on antipsychotic medications.

Clinical studies for treatments that could address redox metabolism include *N*-acetyl-l-cysteine, methylcobalamin, methylcobalamin combined with oral folinic acid and a multivitamin (as previous mentioned). One small open-label study that provided 25–30 μg/kg/day (1500 μg/day maximum) of methylcobalamin to 13 patients found no adverse effects (68) while a medium-sized, open-label trial that provided 75 μg/kg subcutaneously injected methylcobalamin given every 3 days along with twice daily oral low-dose (800 μg/day) folinic acid to 44 children noted some mild adverse effects (69, 70). Four children discontinued the treatment, two because their parents were uncomfortable given injections and two because of hyperactivity and reduced sleep. The most common adverse effect in the participants that remained in the study was hyperactivity, which resolved with a decrease in the folinic acid to 400 μg/day. Lastly, two medium sized, DBPC studies examined *N*-acetyl-l-cysteine, one as a primary treatment and another as an add-on to risperidone. The trial that used *N*-acetyl-l-cysteine as a primary treatment noted no significant differences in adverse events between the treatment and placebo groups, although both groups demonstrated a high rate of gastrointestinal symptoms and one participant in the active treatment phase required termination due to increased agitation (72). In the add-on study, one patient in the active treatment group withdrew due to severe sedation (73). In this latter study, adverse effects were not compared statistically between groups, but most adverse effects were mild and had a low prevalence. Such adverse effects included constipation, increased appetite, fatigue, nervousness, and daytime drowsiness. Lastly, a small DPBC study using vitamin C did not report any adverse effects from the treatment (67). Thus, there are several relatively safe and well-tolerated treatments for addressing abnormal redox metabolism, but there does appear to be a low rate of adverse effects, reinforcing the notion that a medical professional should guide treatment.

Three DBPC studies, one small (110), one medium (111), and one medium-to-large (109) sized, were conducted using sapropterin as a treatment for ASD. None of these studies have reported a higher prevalence of adverse effects in the treatment group as compared to the placebo group and none of these studies attributed any dropouts to the treatment. Thus, sapropterin appears to be a well-tolerated treatment.

## Discussion

One advantage of the treatments outlined above is that the physiological mechanisms that they address are known and biomarkers are available to identify children who may respond to these treatments. Preliminary studies suggest that there are a substantial number of ASD children with these metabolic abnormalities. For example, mitochondrial abnormalities may be seen in 5–80% of children with ASD (31, 43–45, 53, 54) and FRα autoantibodies may be found in 47% (103) to 75% (17) of children with ASD. Clearly, further studies will be required to clarify the percentage of these subgroups.

Further large-scale, multicenter DBPC clinical trials are needed for these promising treatments in order to document the efficacy and define the subgroups that best respond to these treatments. As more treatable disorders are documented and as data accumulates to demonstrate the efficacy of treatments for these disorders, clinical algorithms to approach the work-up for a child with ASD need to be developed by a consensus of experts. Indeed, developing guidelines will be the next step for applying many of these scientific findings. Clearly many children with ASD may be able to benefit from such treatments, which are focused on improving dysfunctional physiology. Given the fact that no approved medical treatment exists which addresses the underlying pathophysiology or core symptoms of ASD, these treatments could make a substantial difference in the lives of children with ASD and their families. With the high prevalence of ASD, treatments that successfully treat even only a fraction of children affected with ASD would translate into substantial benefits for millions of individuals with ASD and their families. In summary, it appears that many of these treatments may provide benefit for a substantial proportion of children with ASD.

## Conflict of Interest Statement

The authors declare that the research was conducted in the absence of any commercial or financial relationships that could be construed as a potential conflict of interest.
